# Virulence Differences Among *Francisella tularensis* Subsp. *tularensis* Clades in Mice

**DOI:** 10.1371/journal.pone.0010205

**Published:** 2010-04-16

**Authors:** Claudia R. Molins, Mark J. Delorey, Brook M. Yockey, John W. Young, Sarah W. Sheldon, Sara M. Reese, Martin E. Schriefer, Jeannine M. Petersen

**Affiliations:** Division of Vector-Borne Infectious Diseases, Centers for Disease Control and Prevention, Fort Collins, Colorado, United States of America; The Research Institute for Children at Children's Hospital New Orleans, United States of America

## Abstract

*Francisella tularensis* subspecies *tularensis* (type A) and *holarctica* (type B) are of clinical importance in causing tularemia. Molecular typing methods have further separated type A strains into three genetically distinct clades, A1a, A1b and A2. Epidemiological analyses of human infections in the United States suggest that A1b infections are associated with a significantly higher mortality rate as compared to infections caused by A1a, A2 and type B. To determine if genetic differences as defined by molecular typing directly correlate with differences in virulence, A1a, A1b, A2 and type B strains were compared in C57BL/6 mice. Here we demonstrate significant differences between survival curves for infections caused by A1b versus A1a, A2 and type B, with A1b infected mice dying earlier than mice infected with A1a, A2 or type B; these results were conserved among multiple strains. Differences were also detected among type A clades as well as between type A clades and type B with respect to bacterial burdens, and gross anatomy in infected mice. Our results indicate that clades defined within *F. tularensis* subsp. *tularensis* by molecular typing methods correlate with virulence differences, with A1b strains more virulent than A1a, A2 and type B strains. These findings indicate type A strains are not equivalent with respect to virulence and have important implications for public health as well as basic research programs.

## Introduction

*Francisella tularensis* is the etiologic agent of the zoonotic disease tularemia and one of the most infectious bacterial pathogens known to humans and animals. The infectious dose of this bacterium is between 10 and 50 CFU in humans depending upon the route of infection [1]. Tularemia can be acquired through the bite of an infected arthropod (ticks, deerflies and mosquitoes), by handling infected animal carcasses, through the ingestion of contaminated food or water, and by inhalation of infective aerosols. Distinct clinical presentations of tularemia occur and are dependent upon the route of infection [1], [2].

Two subspecies of *F. tularensis*, subspecies *tularensis* (type A) and subspecies *holarctica* (type B) are of clinical importance in causing tularemia [3]. Differentiation between these two subspecies is based on biochemical testing, molecular typing methods, virulence, and epidemiological characteristics [3], [4]-[11]. Geographically, *F. tularensis* subsp. *tularensis* infections are documented only in North America, as compared to *F. tularensis* subsp. *holarctica* infections which occur in Europe, Asia and North America [1], [4], [5]. Virulence differences between *F. tularensis* subsp. *tularensis* and subsp. *holarctica* have been demonstrated in rabbits, with one bacterial cell of an *F. tularensis* subsp. *tularensis* strain sufficient to cause 100% mortality as compared to 10^9^ cells required for an *F. tularensis* subsp. *holarctica* strain [4].

Pulsed-field gel electrophoresis (PFGE) and whole genome single nucleotide polymorphism (SNP) typing of *F. tularensis* subsp. *tularensis* strains have subdivided this subspecies into three genetically distinct clades, A1a, A1b and A2 [8], [12], [13]. Historically, *F. tularensis* subsp. *tularensis* strains have been considered to be of similar virulence. However, recent epidemiological analysis of culture-confirmed human infections in the United States showed that infections caused by A1b strains resulted in significantly higher mortality (24%) than infections caused by A1a (4%), A2 (0%) or type B (7%) strains [12]. Logistic regression analysis of A1b infections indicated that this higher mortality rate was not linked to host characteristics, including age or immune status, suggesting that A1b strains have an intrinsic characteristic which makes them more virulent than A1a, A2 or type B strains.

The objective of the present study was to determine if genetic differences, as defined by molecular typing, directly correlate with differences in virulence in infected mice. Mice are not commonly used to compare virulence of *F. tularensis* strains, as early reports indicated no difference in LD_50_ between type A and type B strains [1], [14], [15]. However, Olsufiev *et al*. demonstrated differences in time to death between mice infected with a type A strain (Schu) and a type B strain (503) [4]. More recently, when type A and type B strains were compared using aerosol, intradermal, and intravenous routes of infection in both BALB/c and C57BL/6 mice, type A infected mice succumbed to infection approximately one day earlier than mice infected with type B [16]. A separate study reported significant differences in survival curves between two type A strains (FSC033 and Schu S4) in BALB/c mice [17].

In this study, we compared survival curves, bacterial burden and gross anatomy for mice infected with the three type A clades, A1a, A1b and A2, as well as type B. Significant differences in survival curves (scale and shape) were observed between *F. tularensis* subsp. *tularensis* clades, as well as between these clades and type B, with A1b infected mice dying sooner than A1a, A2 or type B infected mice.

## Materials and Methods

### Bacterial isolates and preparation of inoculum

All *F. tularensis* strains (n = 8) used to infect mice originated from human cases of tularemia (Table 1). Strains were grown from frozen stocks (−70°C) on cysteine heart agar with 9% chocolatized sheep blood (CHAB) at 35°C for 48 h, followed by subculture onto CHAB for 24 h at 35°C. Bacterial suspensions for inoculations were prepared in sterile saline. Colony forming units in each inoculum were verified by spotting 50 µl of the inoculum onto each quadrant of two CHAB quad plates (8 replicates total) and letting the plates dry without spreading. Colony forming units were counted after 48–72 hours of growth at 35°C.

**Table 1:** *Francisella tularensis* strains used in this study.

CDC Accession Number	*Francisella tularensis* Subspecies	Clade	Year Isolated	Geographic Origin	Round Used	Average Inoculum (CFU)
OK01-2528	*tularensis*	A1a	2001	Oklahoma	1	16±3
MO02-4195	*tularensis*	A1a	2002	Missouri	2	11±4
MD00-2970	*tularensis*	A1b	2000	Maryland	1	13±2
MA00-2987	*tularensis*	A1b	2000	Massachusetts	2	10±4
WY96-3418	*tularensis*	A2	1996	Wyoming	1	14±4
NM99-1823	*tularensis*	A2	1999	New Mexico	2	11±3
KY99-3387	*holarctica*	-	1999	Kentucky	1	14±3
MI00-1730	*holarctica*	-	2000	Michigan	2	14±6

### Pulsed-field gel electrophoresis

All *F. tularensis* strains were characterized using *Pme*I pulsed-field gel electrophoresis [8], [12]. PFGE patterns were analyzed by BioNumerics, version 5.0 (Applied Maths, BVBA, Sint-Martens-Latem, Belgium), with gels normalized using *AscI* restricted *Salmonella enterica* serotype Braenderup strain H9812. A dendogram was generated using Dice similarity coefficients and unweighted pair group method with averages (1.5% tolerance, 1.5% optimization).

### Animal infections

Specific-pathogen free female C57BL/6J mice, (The Jackson Laboratory, Bar Harbor, ME) 8–9 weeks of age, were used. For *F. tularensis* infections, mice were anesthetized by inhalation of isoflurane to effect and infected intradermally with 50 µl via the tail dermis. Control mice were inoculated with 50 µl of saline. Seven mice were infected with each strain to ensure sufficient power to detect a scale shift in the fitted survival curve of at least one day. Experiments were performed in two rounds, with each round consisting of mice being infected with one strain for each *F. tularensis* group (A1a, A1b, A2 and type B). Infected mice were monitored every 1–2 hours (minimum of 12 observations per day) until death. Mice were weighed prior to infection and at time of death. Mice were given food ad libitum and an exercise wheel was provided in every cage. All animal procedures were approved by the Division of Vector-Borne Infectious Diseases Institutional Animal Care and Use Committee (protocol number 08–012) and performed in accordance with the guidelines on the care and use of laboratory animals [18]. The temperature of infected mice was also monitored in these experiments in order to identify an ethical, unbiased surrogate endpoint for death (manuscript in preparation). All animal experiments with *F. tularensis* were conducted in ABL3 facilities.

### Quantitative bacteriology

Necropsies were performed at the time of death. Whole organs (lungs, spleen, and liver) were removed aseptically from infected and control mice. Liver and lungs were briefly submerged into sterile saline to remove any traces of contaminating blood, and excess saline was dabbed onto a sterile petri dish. Each organ was weighed and then sterile saline added (5 ml for spleen and lungs and 10 ml for liver) prior to homogenization using a Stomacher 80 micro Biomaster (Seward, Bohemia, NY). Homogenized samples were 10 fold serially diluted in sterile saline up to 10^8^ and serial dilutions (dependent on sample) spotted (50 µl) in duplicates onto CHAB quad plates, with plates allowed to dry without spreading. Colonies were counted after 48–72 hours of growth at 35°C. Whole blood was collected from the abdominal aorta and 10 fold serially diluted into sterile saline to a 10^8^ dilution and serial dilutions, 10^6^, 10^7^ and 10^8^, plated as described above.

### Data and statistical analyses

Empirical survival curves were generated by plotting proportion of surviving mice against time of death (hours). Median time to death was determined using JMP 7 version 7.0.2 software (SAS, Cary, NC). Survival curves were modeled according to a Weibull distribution using S-Plus 8.0 software (TIBCO Software Inc, Palo Alto, CA) allowing different shape and scale parameter estimates for the four groups and two rounds of experiments. Residual plots and goodness of fit tests were used to validate the model fit. To determine if survival curves differed in round 1 and round 2, fitted survival curves for the two strains within each of the 4 groups (A1a, A1b, A2 and type B) were compared across the two rounds of experiments following Lee [19]. A Bonferroni adjustment was made to account for multiple comparisons, with the overall probability of a Type I error being 0.05. Additionally, a survival regression model was fit with terms for interaction between group and round.

For bacterial burden, mouse weight and organ weight comparisons, a weighted 2-way MANOVA was used followed by appropriate tests (either Tukey's method or Fisher's LSD) for multiple comparisons with the overall probability of a Type I error being 0.05. The main effects in the MANOVA were group and round, and an interaction term between the two was also included to determine if the group effect was consistent between rounds.

## Results

### Genotype comparison of *F. tularensis* strains

**Figure 1:**
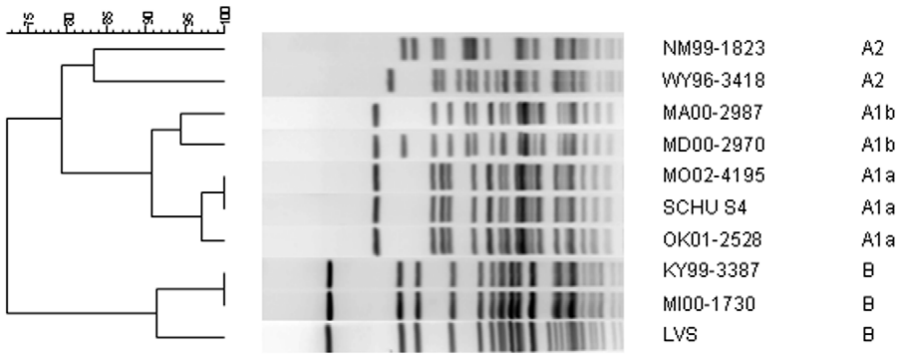
PFGE analysis of *F. tularensis* strains. A diagram depicting the eight *F. tularensis* strains used to infect mice (two strains per each of the four *F. tularensis* groups, A1a, A1b, A2 and type B). *F. tularensis* subsp. *tularensis* strain Schu S4 (A1a) and LVS (type B) were included in the dendogram as reference strains.

*F. tularensis* strains originated from culture-confirmed cases of human tularemia in the United States and were classified into one of four groups, A1a, A1b, A2, or type B, by PFGE typing (Table 1; Figure 1). A total of eight *F. tularensis* strains, two representative strains for each of the four groups, were included (Table 1). Each set of two strains was chosen based on differences in geographical origin and year isolated. Where possible, strains with differing PFGE patterns were chosen to ensure strain diversity within each of the 4 groups (Figure 1).

### Survival comparison of mice infected with *F. tularensis* A1a, A1b, A2 and type B strains

**Figure 2:**
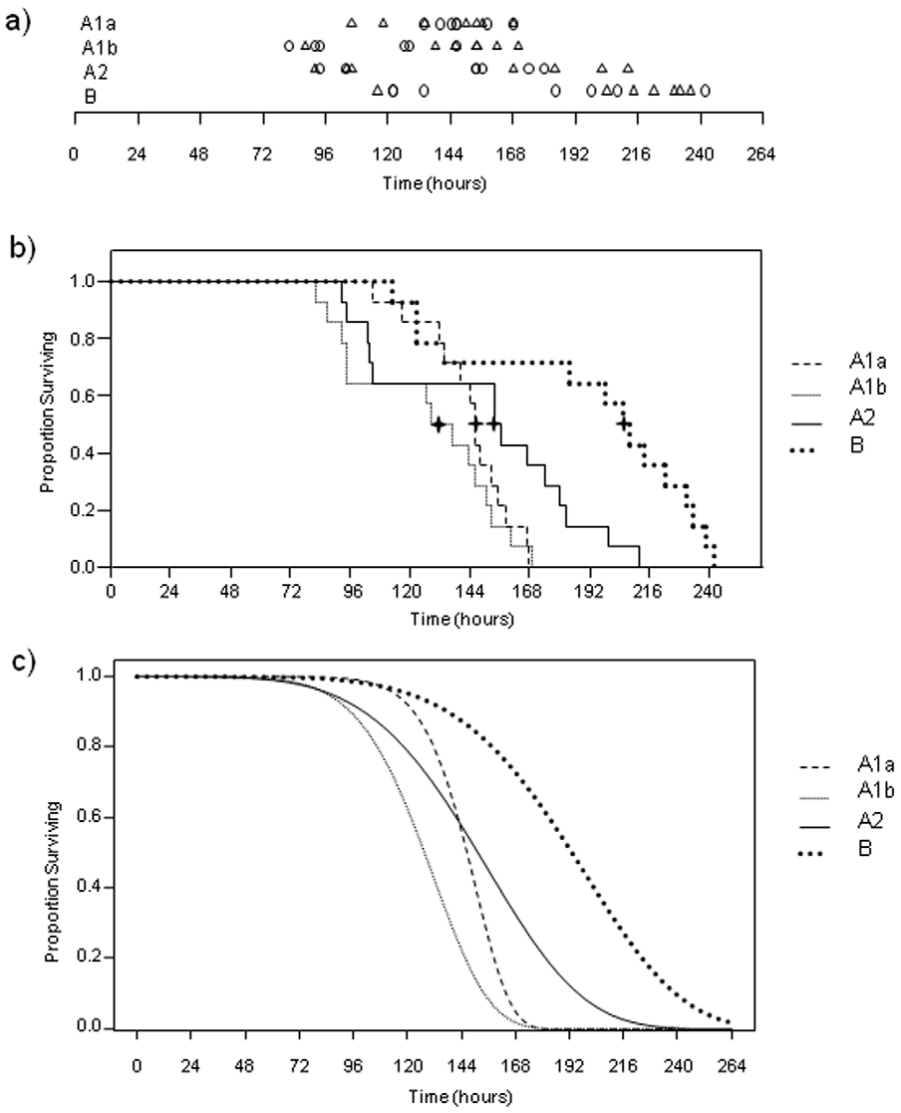
Survival curves of naïve mice following challenge with *F. tularensis* A1a, A1b, A2 and type B. C57BL/6J mice (n = 14/group; n = 7/strain) were challenged intradermally with 10–20 CFU of *F. tularensis* A1a (strains OK01-2528 and MO02-4195), A1b (strains MD00-2970 and MA00-2987), A2 (strains WY96-3418 and NM99-1823) and type B (strains KY99-3387 and MI00-1730), and survival was monitored over time. **A**) Time of death for n = 14 mice per *F. tularensis* group (A1a, A1b, A2 and type B). Circles represent mice infected in round 1(n = 7) and triangles represent mice infected in round 2 (n = 7). **B**) Step-down survival curves for proportion of mice surviving over time (hours). Data shown is from round 1 and round 2 for A1a, A1b, A2 and type B data. Median times of death are noted as a bold cross. **C**) Fitted survival curves for proportion of mice surviving over time (hours) using a Weibull distribution for combined (n = 14 mice/group) round 1 and round 2 A1a, A1b, A2 and type B data.

For each *F. tularensis* strain (n = 8), seven naïve C57BL/6J mice were infected intradermally. At the time of infections, colony counts within each inoculum were determined in octuplicate, with the average colony forming units per inoculum ranging from 10 to16 (Table 1). The time of death for A1a, A1b, A2, and type B infected mice ranged from 117 h to168 h, 82 h to169 h, 92 h to 212 h, and 113 h to 242 h, respectively (Figure 2a). Median times to death were 146 h, 132.5 h, 155 h, and 206.5 h for A1a, A1b, A2 and type B infected mice, respectively (Figure 2b). The median time to death for mice infected with A1b strains was 14, 22, and 74 hrs earlier than mice infected with A1a, A2 or type B strains, respectively.

A regression was performed on all 8 strains with survival time as the response and assuming a Weibull distribution for survival times. In the regression, both the scale and shape parameters of the Weibull distribution were expressed as linear functions of *F. tularensis* group and experimental round (e.g. round 1 or round 2). The interaction term between group and round was not statistically significant (at the α = 0.05 level of significance) for either the scale parameter (p = 0.06) or the shape parameter (p = 0.49). The linear term for round was also not statistically significant for either the scale parameter (p = 0.08) or the shape parameter (p = 0.38). These results suggest that neither of the parameter values differ from round 1 to round 2. Additionally, when individual survival curves were fit to each of the eight sets of data (assuming a Weibull distribution for survival times), none of the scale or shape parameters within group were statistically different (all p-values <0.025). Therefore, round 1 and round 2 data were combined for the two strains constituting each *F. tularensis* group (Figure 2c).

For comparisons between *F. tularensis* infection groups, differences were considered statistically significant if p<0.008, with this level of significance determined using the Bonferroni adjustment for multiple comparisons. Comparison between *F. tularensis* groups indicated that the survival curve for A1b infected mice differed significantly (p<0.008) in scale parameter as compared to survival curves for A1a, A2 and type B infected mice. The shift of the curve reflected that mice infected with A1b died earlier than mice infected with A1a, A2 or type B (Figure 2c). The survival curves for mice infected with A1a and A2 also differed significantly in scale from the survival curve for mice infected with type B (p<0.008) (Figure 2a and 2c). Additionally, the survival curve for mice infected with A1a differed significantly (p<0.008) in shape from the survival curves for mice infected with A1b, A2 and type B, reflecting that mice died within a narrower timeframe when infected with an A1a strain as compared to mice infected with A1b, A2 or type B (Figure 2a). The survival curve for mice infected with A1a did not differ significantly in scale from the survival curve for mice infected with A2 (0.05<p<0.10).

### Bacterial burdens in mice infected with *F. tularensis* A1a, A1b, A2 and type B strains

**Figure 3:**
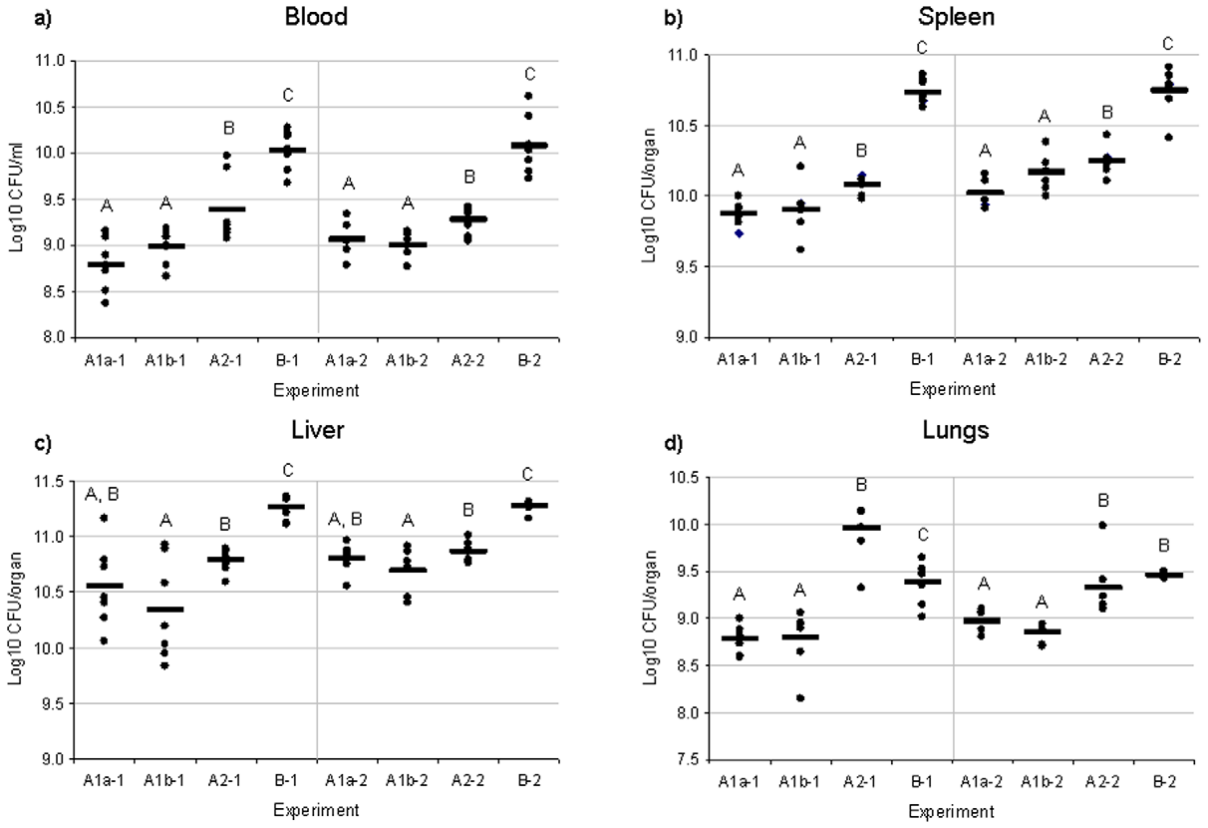
Bacterial burden of *F. tularensis* within the blood, spleen, liver and lungs of infected mice. C57BL/6J mice (n = 14/group) were challenged intradermally with 10-20 CFU of *F. tularensis* A1a, A1b, A2, and type B. At time of death, organs were harvested and blood was taken from each mouse. **A**) Bacterial burden (Log_10_ CFU/ml) within blood samples of all infection groups. No statistical difference was observed between round 1 and round 2. **B**) Bacterial burden (Log_10_ CFU/spleen) within spleens of all infection groups. A statistical difference was observed between round 1 and round 2. **C**) Bacterial burden (Log_10_ CFU/liver) within livers of all infection groups. No statistical difference was observed between round 1 and round 2. **D**) Bacterial burden (Log_10_ CFU/lung) within lungs of all infection groups. A statistical difference was observed between round 1 and round 2. For all graphs, significant differences (p<0.05) in Log_10_ CFU within round 1 infections and within round 2 infections are shown by different letters (A, B, etc.). If two strains have the same letter, no significant differences were identified between them.

To determine if differences in bacterial loads occurred among mice infected with *F. tularensis* groups (A1a, A1b, A2, and type B), bacterial burdens were determined at time of death and compared among groups for blood, spleen, liver and lungs (Figure 3). Comparisons were performed using an overall Type I error rate of α = 0.05, thus statistically significant results have an adjusted p value <0.05. Mean bacterial load in the blood and liver did not differ significantly between rounds for any two strains of the same *F. tularensis* group. Mean bacterial load in the spleen was higher in round 2 (p<0.01). The increase in bacterial burden within the spleen was consistent for all groups and the ordering of groups by bacterial load was preserved from round 1 to round 2. The interaction term between group and round was statistically significant (p<0.01) for bacterial burdens in the lungs. This suggests that the magnitude of differences in bacterial burdens for lungs among the groups is not the same from round 1 to round 2.

As shown in Figure 3, our results indicated that the mean bacterial load in both blood and spleen was statistically highest in type B infected mice. The second highest bacterial load in the blood and spleen was in A2 infected mice, which was statistically higher than that of either A1a or A1b infected mice. No statistical difference in mean bacterial load was detected between A1a infected mice and A1b infected mice in either blood or spleen tissue. The estimated mean Log_10_ CFU/ml blood was 8.73, 9.08, 8.99, 9.01, 9.38, 9.27, 10.03 and 10.09 for A1a-1, A1a-2, A1b-1, A1b-2, A2-1, A2-2, B-1 and B-2 infected mice, respectively. The estimated mean Log_10_ CFU/spleen was 9.87, 10.02, 9.91, 10.16, 10.08, 10.25, 10.74 and 10.75 for A1a-1, A1a-2, A1b-1, A1b-2, A2-1, A2-2, B-1 and B-2 infected mice, respectively.

In the liver, mean bacterial burden was also statistically highest in type B infected mice (estimated mean Log_10_ CFU/liver of 11.27 for both B-1 and B-2). Mean bacterial burden in A2 infected mice (Log_10_ CFU/liver of 10.78 and 10.86 for A2-1 and A2-2, respectively) was statistically higher than in A1b infected mice (Log_10_ CFU/liver of10.34 and 10.70 for A1b-1 and A1b-2, respectively) but not higher than A1a infected mice (Log_10_ CFU/liver of 10.55 and 10.83, for A1a-1 and A1a-2, respectively). There was no statistical difference in mean bacterial burden in the liver between A1a and A1b infected mice.

Mean bacterial loads in the lungs were statistically higher in A2 (estimated mean Log_10_ CFU/liver of 9.95 and 9.32 for A2-1 and A2-2, respectively) and type B (estimated mean Log_10_ CFU/liver of 9.38 and 9.47 for B-1 and B-2, respectively) infected mice as compared to A1a (estimated mean Log_10_ CFU/liver of 8.82 and 8.97 for A1a-1 and A1a-2, respectively) and A1b (estimated mean Log_10_ CFU/liver of 8.80 and 8.85 for A1b-1 and A1b-2, respectively) infected mice. In round 1, mean bacterial load was statistically higher in A2 infected mice than in type B infected mice. No statistically significant differences in lung bacterial burden were detected between A2 and type B infected mice in round 2 or between A1a and A1b infected mice in either round.

### Gross anatomy of mice infected with *F. tularensis* A1a, A1b, A2, and type B strains

**Figure 4:**
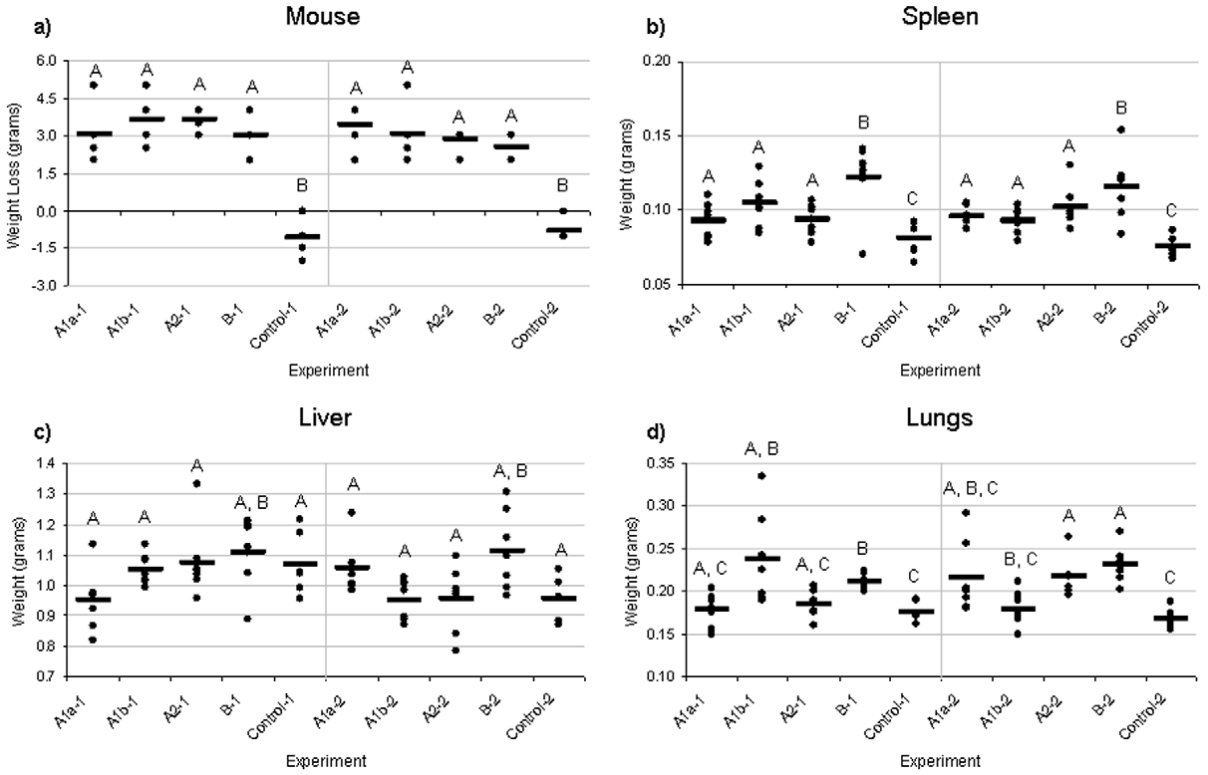
Weight loss and spleen, liver and lung weights for mice infected with *F. tularensis* A1a, A1b, A2, and type B. C57BL/6J mice (n = 14/group) were challenged intradermally with 10-20 CFU of *F. tularensis* A1a, A1b, A2, and type B. Mice were weighed prior to infection and at time of death. Organs were harvested and weighed at time of death. **A**) Mouse weight (grams) for each infection group and both control groups. No statistical difference was observed between round 1 and round 2. **B**) Spleen weight (grams) for each infection group and both control groups. No statistical difference was observed between round 1 and round 2. **C**) Liver weight (grams) for each infection group and both control groups. No statistical difference was observed between round 1 and round 2. **D**) Lung weight (grams) for each infection group and both control groups. A statistical difference was observed between round 1 and round 2. For all graphs, significant differences (p<0.05) in Log_10_ CFU within round 1 infections and within round 2 infections are shown by different letters (A, B, etc.). If two strains have the same letter, no significant differences were identified between them.

To determine if differences in gross anatomy occurred among mice infected with *F. tularensis* A1a, A1b, A2, and type B strains, the weight of each mouse and its spleen, liver and lungs were measured at time of death (Figure 4). Multiple comparisons were done with an overall Type I error rate of α = 0.05, thus statistically significant results have an adjusted p value <0.05. No significant differences were found in mouse weight between any of the infection groups (p = 0.07) or rounds (p = 0.06). However, weight loss was statistically lower in the control group compared to the infection groups. Mice infected with all *F. tularensis* strains had an overall average weight loss of 2.6 to 3.6 g as compared to non-infected mice, with A2 and type B infected mice losing the least amount of weight (Figure 4).

Mean spleen weight was found to be statistically higher in type B infected mice than in any of the type A infected mice, while mean spleen weight was statistically lowest in the control group. Mean liver weight was statistically higher in type B infected mice than in A1a infected mice in round 1 and was statistically higher in type B infected mice than A1b infected mice in round 2. No other statistical differences in mean liver weight by group or round were detected. There was an inconsistent pattern in mean lung weight between rounds 1 and 2. In round 1, A1a infected mice had a statistically lower mean lung weight than A1b and type B infected mice. Additionally, A2 infected mice had a statistically lower mean lung weight than type B infected mice. In round 2, A1b infected mice had a statistically lower mean lung weight than either A2 or type B infected mice. The control group had a statistically lower mean lung weight than A1b infected mice and type B infected mice in round 2.

Gross changes in the liver and spleen were also identified at time of death. Spleens and livers were noticeably enlarged in type B infected mice as compared to non-infected mice or mice infected with A1a, A1b or A2 strains and showed less discoloration than those from mice infected with A1a, A1b or A2 strains (data not shown). Additionally, tissue damage, both necrosis and granulomas, were apparent in spleens of mice with A1a, A1b or A2 as opposed to type B infected mice (data not shown).

## Discussion

In this study, we demonstrate that genetic differences (clades A1a, A1b and A2) among *F. tularensis* subsp. *tularensis* strains, as identified by PFGE and SNP typing, correlate directly with differences in virulence as assessed using C57BL/6 mice. Among type A clades, A1b strains were shown to be the most virulent in mice, with A1b infected mice succumbing to infection at significantly earlier times as compared to A1a and A2 infected mice. Additionally, A1a infected mice died within a narrower time span than A1b or A2 infected mice, suggesting that A1a strains are either more clonal than A1b or A2 strains or utilize a different mechanism for infection. Importantly, virulence differences appeared to be conserved among clades, as two representative strains with slightly differing PFGE patterns from each type A clade, A1b, A1a and A2, were utilized in this study. Virulence differences were also demonstrated between all type A clades and type B.

Measurement of bacterial burden within the blood, spleen and liver at the time of death in mice infected with A1a, A1b, A2 and type B strains indicated a significantly higher bacterial load in type B-infected mice as compared to mice infected with any of the type A clades. Similarly, mice infected with A2 strains had significantly higher bacterial loads in the blood and liver as compared to A1a and A1b infected mice. A correlation between bacterial burden and time to death was observed, with mice succumbing to infection earlier (i.e. A1a and A1b infected mice) having lower bacterial burdens than mice dying at later time points (i.e. A2 and type B infected mice). This data demonstrates that higher bacterial loads are needed for type B and A2 infected mice to die from infection, as compared to A1a or A1b infected mice. It is unknown if differences in growth rates exist between type A clades (A1a, A1b and A2) or between type A clades and type B. In addition to type B infected mice having higher bacterial burdens, organs were larger in type B infected mice, indicative of a strong inflammatory response that is not as pronounced in type A infected mice.

Our findings are in agreement with a study performed by Twine *et al.*, which demonstrated virulence differences between two type A strains, Schu S4 and FSC033, in BALB/c mice [17]. Although significant differences in survival curves were found between these two strains, with FSC033 appearing more virulent than Schu S4, they were not attributed to genetic differences. PFGE typing demonstrates that Schu S4 is an A1a strain (Figure 1), whereas analysis of the genome sequence for FSC033 shows it contains single nucleotide polymorphisms characteristic of A1b strains (C at position 93857 in the FSC033 genome using primer set 1574929 and a G at position 5357 in the FSC033 genome using primer set 518892) [13].

Due to the low infectious dose of *F. tularensis* for both type A and type B in mice (LD_50_ <10 CFU for both subspecies) and the acute and rapid onset of infection that results, few studies have used a mouse model to demonstrate tangible virulence differences between *F. tularensis* strains [1], [4], [14], [15]. Nonetheless, our results indicate that survival curves can be used to assay differences in virulence in mice among both type A clades and subspecies of *F. tularensis*. Several factors are likely to be critical for identifying virulence differences among *F. tularensis* strains using a mouse model. In this study, we used death as an endpoint to generate survival curves, thereby eliminating any bias in using signs of morbidity to euthanize mice and determine time of death. Towards this end we have defined a specific temperature during infection that is an ethical indicator of death (unpublished observation). In the future, the use of temperature as an experimental endpoint will minimize pain and suffering of infected animals and will also serve as a valuable tool for unbiased comparisons of *F. tularensis* virulence. Monitoring mice every 2 hours over the time course of infection, rather than 24 hr periods, was also important in this study for ensuring accuracy. Finally, genotyped clinical strains were chosen for this study in order to yield information directly related to previous epidemiologic findings [12].

The mechanisms underlying virulence differences among type A clades and between type A clades and type B are not known. To date, numerous studies have compared genetic variations present between type A and type B strains at both the genome and protein level and a few studies have also compared differences between type A clades, A1 and A2 [10], [20]–[24]. A previous study showed that FSC033 was better able to disseminate as compared to Schu S4 from the original inoculation site to the liver and spleen of infected mice [17]. Consistent with A1b strains being better able to disseminate is the epidemiologic finding that the majority of isolates (67%) from patients infected with A1b strains were recovered from invasive sites (blood, lung, pleural fluid or CSF) as compared to patients infected with A1a (44%), A2 (10%) or type B (37%) strains [12]. Rates of dissemination, immunological responses, and tissue tropism are areas for future analyses.

In conclusion, our results correlate with epidemiological data for human culture-confirmed tularemia cases in the United States, which demonstrated that A1b infections were associated with higher mortality as compared to A1a, A2 and type B infections [12]. Here we showed that mice infected with A1b strains had a significantly shortened survival time as compared to mice infected with A1a, A2 or type B strains, demonstrating the importance of human epidemiological data and how it can yield hypotheses that can be studied in the laboratory. This finding will be important for raising awareness in states where human infections with A1b strains occur, which may aid in preventing mortality. The virulence differences identified among type A clades indicate we can no longer consider *F. tularensis* subsp. *tularensis* to be biologically homogeneous and the need to shift our attention from the subspecies to the clade level. Previous studies also showed differences in geographic distributions among type A clades [8], [20]. The demonstration of genetic and geographic, as well as virulence, differences among the A1a, A1b and A2 clades brings into question their classification, given that *F. tularensis* subsp. *tularensis* was originally classified as a subspecies based on biochemical, geographic and virulence differences [3].
